# Pitch as the Main Determiner of Italian Lexical Stress Perception Across the Lifespan: Evidence From Typical Development and Dyslexia

**DOI:** 10.3389/fpsyg.2019.01458

**Published:** 2019-06-26

**Authors:** Martina Caccia, Giorgio Presti, Alessio Toraldo, Anthea Radaelli, Luca Andrea Ludovico, Anna Ogliari, Maria Luisa Lorusso

**Affiliations:** ^1^Center for Neurocognition, Epistemology and Theoretical Syntax, University School For Advanced Studies, Pavia, Italy; ^2^Unit of Neuropsychology of Developmental Disorders, Department of Child Psychopathology, Scientific Institute IRCCS “E. Medea”, Bosisio Parini, Italy; ^3^Laboratory of Music Informatics (LIM), Department of Computer Science, University of Milan, Milan, Italy; ^4^Department of Brain and Behavioural Sciences, University of Pavia, Pavia, Italy; ^5^Milan Center for Neuroscience, Milan, Italy; ^6^Developmental Psychopathology Unit, Vita-Salute San Raffaele University, Milan, Italy

**Keywords:** lexical stress, acoustic parameters, developmental dyslexia, developmental trajectories, Italian language, pitch, duration, intensity

## Abstract

The study deals with the issue of lexical stress perception in both a developmental (comparing children and adults with typical development) and a clinical perspective (comparing typically developing children and children with dyslexia). The three parameters characterizing the acoustic profiles of words and non-words in a certain language are duration, pitch and intensity of its syllables. Based on (sparse) previous literature on Italian and other European languages, it was expected that syllable duration would be the parameter predominantly determining the perception of stress position. It was furthermore anticipated that children with dyslexia may be found to have an altered perception of lexical stress, due to their impairments in auditory processing of either pitch, duration or (more controversial) intensity. Systematic manipulation of the pitch, duration and intensity profiles of three Italian trisyllabic non-words produced a series of 81 stimuli, that were judged with respect to stress position (perceived on the ultimate, penultimate, or antepenultimate syllable) by the three groups of participants. The results showed, contrarily to expectations, that the pitch component is the most reliable acoustic cue in stress perception for both adults, in whom this dominance is very strong, and typically developing children, who showed a similar but quantitatively less marked pattern. As to children with dyslexia, they did not seem to rely on any parameter for their judgments, and rather gave random responses, which point to a general inability to process the various acoustic modulations that normally contribute to stress perception. Performance on the stress perception task strongly correlates with language (morphosyntactic) measures in the whole sample of children, and with reading abilities in the group with dyslexia, confirming the strict relationship between the two sets of skills. These findings seem to support a language-specific approach, suggesting that the set of acoustic parameters required for the development of stress perception is language-dependent rather than universal.

## Introduction

Stress is an important prosodic feature which “makes one syllable in a word more prominent than its neighbors” (Himmelmann and Ladd, 2008, p. 248). Stress contributes to create rhythm in speech and each language is characterized by its own rhythmic pattern (Kuhn et al., 2010); rhythm can be defined as the alternation of strong and weak beats recurring in the sequence of auditory events (Huss et al., 2011).

Languages differ not only in their segmental possibilities, but also in their use of prosodic cues to convey differences in meaning. For example, tone languages, such as Chinese, use variations in pitch to distinguish among different lexical items. These pitch differences seem to be difficult to perceive for adult speakers of non-tonal languages such as English (Wang et al., 1999). Moreover, in a cross-linguistic study, Dupoux et al. (1997) showed that native speakers of French have more difficulties in perceiving word stress than native speakers of Spanish. Indeed, Spanish uses stress in a contrastive way (to distinguish between words) but French does not. The authors conclude that French speakers probably use stress at a different level, for instance for finding word or phonological phrase boundaries.

Following the rhythm detection hypothesis (RDH) (Goswami et al., 2002, 2011), phonological development seems strictly driven by the sensitivity to slower rather than rapid auditory events. However, Antoniou et al. (2015) suggested that the set of acoustic cues required for language development is language-specific (LSAC, language-specific auditory cue) rather than universal as postulated by RDH. Specifically, tone languages, such as Chinese, seem to be based on pitch movement (the rise and fall of the pitch) (Chung et al., 2017); actually, pitch contour sensitivity (or sensitivity to intonation) appears to be fundamental to phonological, reading and language development both in Mandarin (Goswami et al., 2011) and Cantonese (Antoniou et al., 2015).

The issue of universal vs. language-specific phenomena in stress perception had already been addressed within other conceptual frameworks, such as studies concerning the so-called P-center. Morton et al. (1976) defined the “perceptual center” or “P-center” as the perceptual moment of occurrence of a monosyllabic token. Indeed, languages greatly differ with respect to their rhythmic organization. It has been proposed that they can be subdivided into three classes: stress-timed (e.g., English and German), syllable-timed (e.g., French and Spanish), and mora-timed (Japanese). In stress-timed languages, which are by far the most studied ones, the intervals between stressed syllables should be approximately isochronous. However, research has failed to confirm strict isochrony between acoustically defined intervals in speech produced in various conditions (Lehiste, 1977; Fox, 1987). Thus, the perception of rhythmicity does not seem to arise from the presence of isochronous acoustic onsets of linguistic elements such as stressed syllables, nor is it easily amenable to any other common measures of acoustic energy. In stressed syllables, it can be affected by the duration of the single vowels and consonants, the presence of unstressed prefixes and/or suffixes (Fox and Lehiste, 1985, 1987), or vowel onset (Fowler, 1983). In Czech disyllabic words, also the number of consonants, as well as some speaker-related abilities were found to influence the position of the P-center (Šturm and Volín, 2016). Thus, the phonetic structure of the whole word may contribute to the P-center location. Hoequist (1983) examined the P-center effect in the production of English, Spanish, and Japanese monosyllables and showed a significant Onset Type effect (same vs. different) but no specific Language effects nor any interactions with language, thus leading to the conclusion that the P-center effect is a universal phenomenon.

Fox (1987) also investigated whether the perceived location of the P-center is generalizable across different languages, comparing monolingual Japanese and American English speakers and came to the conclusion that, for American English, vowel duration causes a shift in the P-center location, as a function of the final consonant duration. This would be true also for Japanese speakers, although the absolute values of the parameters were not identical, and moreover, the contribution of the final consonant was irrelevant. These results support the hypothesis that, in spite of some minor differences in timing between languages, the P-center effect may be common to all (or at least many) languages. However, and most crucially, not much is known concerning more complex syllabic structures and multisyllabic structures following highly language-specific stress rules. Moreover, reported data mainly concern stress-timed and mora-timed languages (Czech being half-way between a stress—timed and a syllable-timed language, depending on the type of NP, see Šturm and Volín, 2016). For these reasons, a study on Italian would provide interesting information about syllable-timed languages.

Indeed, some authors propose that the phonological characteristics of languages could be more relevant than simply isochrony or other forms of temporal organization (Nespor et al., 2011). It may be relevant to observe that different groups of languages are characterized by different amounts of variation: “syllable-timed” languages have a smaller variety of syllable types than “stress-timed” languages, and their syllables are more similar to each other in duration (Dauer, 1983). In Italian, 60 percent of the syllable types are of the CV-type (Bortolini, 1976). Thus, the speakers/listeners of this language may use other cues beyond duration to support stress perception.

Sensitivity to stress patterns is particularly relevant in language learning as it helps the initial segmentation of words from continuous speech (e.g., Mattys et al., 1999) and it also makes information available about the syntactic category of a word. Specifically, stress may allow to discriminate between content words (stressed) and function words (unstressed) (Gleitman and Wanner, 1982) but also between different content words, such as nouns (stress on the first syllable) and verbs (stress on the second syllable) in many languages (Kelly, 1988).

The first studies on word stress perception in Italian suggested that duration, intensity and pitch all contribute to stress assignment (Panconcelli-Calzia, 1912). Gemelli (1950) was the first author who proposed a hierarchy of the acoustic parameters that concur to stress perception: (1) duration, (2) pitch, and (3) intensity. Duration has later been confirmed to be the most reliable cue in stress perception firstly in disyllabic words (Ferrero, 1972; Fava and Magno-Caldognetto, 1976). Always using disyllabic words, Bertinetto (1980) proposed the hierarchy (1) duration, (2) intensity, and (3) pitch in perception. Subsequent studies on disyllabic and trisyllabic words confirmed – with minor differences – the relevance of duration as the most reliable acoustic stress cue in Italian in comparison to other languages (even though the authors specified that duration alone is not sufficient to clearly define stress assignment) (e.g., D’Imperio and Rosenthal, 1999; Alfano, 2006; Alfano et al., 2007; Eriksson et al., 2016). Nonetheless, fundamental frequency seems to play a crucial role in lexical stress perception in English and French (Fant et al., 1991; Hasegawa and Hata, 1992) and in both Japanese and Chinese (Hasegawa and Hata, 1992; Antoniou et al., 2015).

Linguistic prosody seems to play a crucial role in enhancing the perception of single sounds in children’s phonological representations during speech processing (Chiat, 1983; Pierrehumbert, 2003). Consequently, awareness of prosodic patterns (such as English stress and Mandarin tone/pitch) might be important to detect segmentation cues from sound and, therefore, in reading acquisition because children might use such patterns as segmentation cues to sound out words (Chung et al., 2017). Indeed, prosodic awareness seems to be an early ability since many studies have shown that infants are able to perceive the acoustic correlates of word stress from birth. Thus, Italian newborns have been reported to discriminate different stress patterns in di- and trisyllabic pseudo-words (e.g., /’takala/ vs. /ta’kala/), and in lists of pseudo-words with consonantal variation (/’daga ‘nata / vs. /da’ga na’ta /) (Sansavini et al., 1997). Similarly, 2-months-old English infants can discriminate the stress patterns of disyllabic pseudo-words (/’bada’gada/ vs. /ba’da ga’da/) (Jusczyk and Thompson, 1978).

Infants exposed to a language (such as Spanish) with contrastive lexical stress (i.e., with stress-syllable placement determining word meaning) have to process stress patterns not only at the acoustic level, but also at a more abstract, phonological level, since stress could be located on more than one vowel, depending on the specific meaning (Skoruppa et al., 2009). Other studies suggest that stress perception at this abstract level may evolve very early in infant development (Jusczyk et al., 1993). As to English, it is only at 9 months of age that a preference for the predominant stress-initial pattern typical of this language emerges. Moreover, a cross-linguistic study on tone perception in infants shows that between 6 and 6 months of age, English infants’ discrimination abilities for stress perception decline compared to those of Chinese infants (Mattock and Burnham, 2006).

Skoruppa et al. (2009) found that language-specific differences in the perception of stress likewise arise during the first year of life. Specifically, 9-month-old Spanish infants successfully distinguish between stress-initial and stress-final pseudo-words, while French infants of the same age show no sign of discrimination.

Sensitivity to stress patterns seems to be related to the development of skilled reading (Goswami et al., 2002; Orsolini et al., 2006; Wood, 2006 among others) and to reading-related disorders, specifically, developmental dyslexia.

Developmental dyslexia (DD henceforth) is a neurobiological condition with a genetic basis (Siegel and Lipka, 2008; Peterson and Pennington, 2012) that “is manifested in a continuum of specific learning difficulties related to the acquisition of basic skills in reading, spelling and/or writing, such difficulties being unexplained in relation to an individual’s other abilities and educational experiences” (Report of the Task Force on Dyslexia, 2001).

The presence of a deficit at the phonological level and its role in reading disorders are well established. What is still under debate is the nature of these difficulties. Some researchers proposed that besides phonological impairments there is a more basic auditory deficit. Tallal (1980) demonstrated that children with a specific reading impairment face difficulties in making discrimination or temporal order judgements with either very brief tones or tones presented at short (<400 ms) interstimulus intervals (ISIs). In light of the above, Tallal suggested that dyslexic children could have a non-linguistic deficit in temporal resolution of short and rapidly changing auditory stimuli that affects speech perception. Frey et al. (2018) investigated discrimination of phonetic features (syllables differing for voicing, place and mode of articulation) in noise, envelope and silence conditions, and found that children with DD showed longer RTs than their control group across all conditions although they did not differ from TD children in terms of accuracy. The authors proposed that the deficits found in silence conditions might support the hypothesis that internal neural noise disturbs the processing of the acoustic properties of stimuli in dyslexia.

A systematic review on basic auditory processing in DD by Hämäläinen et al. (2013) showed that rise time (meant as the time taken by a signal to change from sound beginning to its maximum amplitude), slow frequency modulation (FM) rates, frequency discrimination with differences smaller than 10%, amplitude modulation (AM) and duration discrimination were most often impaired in individuals with dyslexia (with differences emerging depending on the age of participants and the characteristics of the stimuli or procedures), whereas less consistent findings were found for intensity discrimination and gap perception, that turned out to be unimpaired in dyslexia in most studies.

A number of studies on pitch processing suggest that pitch memory may not be as durable for children as for adults. These studies found declines in children’s memory over the course of a few seconds (Keller and Cowan, 1994; Gomes et al., 1999; see also Trehub et al., 1984). In a behavioral study, Keller and Cowan (1994) showed that 6–7 years old children showed a faster accuracy decrease in a pitch change detection task with variable ISIs compared to adults. Furthermore, several studies showed that pitch processing is sensitive to language experience (e.g., Chinese speakers are more sensitive to pitch variations than English speakers). McAnally and Stein (1996) showed that individuals with DD are impaired in detecting audible changes of tone and in generating phase-locked discharges while decoding pitch variation. Furthermore, Baldeweg et al. (1999) found abnormal mismatch negativity (MMN) during passive pitch discrimination in adults with DD but a normal MMN to tone duration deviants; at the behavioral level, they found an impairment in discriminating tone frequency, but not tone duration. The pitch discrimination and MMN deficit were correlated with the degree of impairment in word and non-word reading accuracy.

On the other hand, Cantiani et al. (2009) and Lorusso et al. (2014) found that children with DD were impaired in temporal processing tasks concerning duration discrimination, in a task requiring discrimination between two rhythms differing for the interval between identical repeated tones. Discrimination of patterns of tones differing in their inter-stimulus intervals had already been found by Schulte-Körne et al. (1999) and Kujala et al. (2000) to differentiate between dyslexic and non-dyslexic participants at the psychophysiological response level.

Many studies have shown that children with DD are impaired in processing rhythmic structures; specifically, they show a lack of sensitivity in lexical stress perception (Goswami et al., 2013) which seems to characterize also adults with reading impairments. Barry et al. (2012) tested the sensitivity to lexical stress in adult German-speaking students with a reading deficit, and found that students with reading problems, despite having normal implicit knowledge of lexical stress rules, failed to show explicit metalinguistic awareness of them. Moreover, children (Goswami et al., 2002, 2010) and adults with DD (Law et al., 2014) show atypical processing of sound rise times and intensity. Studies on Finnish and English showed that the perception of duration in speech sounds is critical in DD (Leppänen et al., 1999, 2002; Richardson et al., 2004). In the Finnish language, duration plays a crucial role in differentiating words both orthographically and semantically; indeed Hämäläinen et al. (2009) found that Finnish-speaking children with DD differed from TD children in duration discrimination but not in the perception of intensity modulation and rise time. Moreover, Ziegler et al. (2012) found that DD show a deficit in pitch contour perception.

Wang et al. (2012) found that in children with DD, accurate discrimination of variation in intensity and rise time was a significant predictor of reading accuracy in Chinese, even if intensity discrimination was not found to be an important source of inter-individual differences in many alphabetic languages (Muneaux et al., 2004; Richardson et al., 2004; Goswami et al., 2010; Hämäläinen et al., 2013). Furthermore, Wang et al. (2012) found that duration and frequency discrimination contribute significant unique variance to tasks of onset and rhyme awareness.

Stress assignment in Italian polysyllabic words is neither diacritically marked nor predicted by rules. Most three- and four-syllable words are stressed on the penultimate syllable, which is considered as the dominant (or “regular”) stress. A smaller proportion of polysyllabic words are stressed on the antepenultimate syllable (non-dominant or “irregular” stress; e.g., Toraldo et al., 2006). Even if the knowledge of distributional properties of sound–spelling mappings is acquired quite early, it could vary as a function of age and reading/spelling experience, also in consistent orthographies like Italian. Indeed Angelelli et al. (2010) and Paizi et al. (2011) showed that children with DD performed very poorly with low-frequency words, indicating a possible lack or unavailability of orthographic representations for this kind of material; they also highlighted a reduced lexical processing ability compared to control readers in both spelling and reading tasks in Italian. Moreover, children with specific learning disabilities tend to omit the Italian diacritical stress, which is compulsory for Italian words with stress on the last syllable. However, these children also proved able to take into account the distributional properties of Italian sound–spelling mappings. This effect was present in both reading and spelling, although with notable differences as a function of word frequency. The distributional properties of sound–spelling mappings were detected by third grade, indicating early acquisition of this skill even in children with dyslexia and dysgraphia (Marinelli et al., 2017).

In the present study, the reliability of duration, pitch and intensity as predictors of stress perception was investigated both in a developmental and in a clinical perspective. To our knowledge, there are no previous studies that investigated the role of the acoustic cues in Italian lexical stress perception in both children – typically and atypically developing – and adults. In order to avoid effects due to familiarity, frequency and other lexical variables, only non-words were used in the study. Furthermore, different types of syllable were employed so as to have a larger variety of stimuli and a representative sample of the typical repertoire of Italian lexical strings. Moreover, three-syllabic non-words were considered, so as to have information on three possible stress positions in Italian words: antepenultimate – AP, penultimate – PE, and ultimate – U, with stress on the first, the second and the third syllable, respectively.

The critical manipulation was the dissociation of the three relevant parameters, duration, intensity, and pitch, from one another. By means of a dedicated software, we could build a balanced set of new acoustic stimuli which vary, independently, for the duration profile (AP, PE, U), the intensity profile (AP, PE, U) and the pitch profile (AP, PE, U). For instance, in the set we had a stimulus whose duration profile was that of an AP stimulus, whose intensity profile was that of an U stimulus and whose pitch profile was that of a PE stimulus. All possible combinations were used, and allowed us to derive “consistency” scores, expressing to what degree a given participant used duration, or intensity, or pitch, to determine his/her perceived stress position.

Based on the analysis of previous literature, we expected that:

(i) in general, duration should be the critical parameter in determining stress assignment;
(ii) children should not differ from adults in the perception and use of acoustic parameters;
(iii) children with DD should be less sensitive than typically developing peers to changes in the acoustic parameters while processing stress position. More specifically, it was expected that use of cues based on duration and pitch would be impaired in DD, whereas intensity would not.

## Materials and Methods

### Participants

Typically developing children (TD), children with dyslexia (DD) and normotypical adults participated in this study. Selection criteria are detailed below.

*Children with DD* were selected among those diagnosed at the Scientific Institute “E. Medea” or at the clinical services depending on San Raffaele-Ville Turro hospital as having Specific Reading Disorders according to standard ICD-10 criteria (World Health Organization, 1992). We included in the study only children who had a score at least 2 SD below the mean in at least two reading tests (speed and/or accuracy parameters), and an IQ score ≥ 80 (see later for further details).

*TD children* were recruited from local primary schools. As a selection criterion, these children were administered with a battery of tests assessing their general intellectual and linguistic abilities (see list below). Children who scored more than 1.5 SD below the mean in at least one test were excluded from the study.

*Normotypical adults* were recruited among the experimenters’ acquaintances and students at S. Raffaele University. Participants with self-reported hearing impairments, learning disabilities and previous language impairments were excluded.

Before starting the experimental task, children of both groups (TD and DD) were asked to carry out a stress-perception test in order to ascertain if they were familiar with the task of identifying stress position. The pre-test consisted of a list of 24 trisyllabic Italian words with different stress position (antepenultimate – AP, penultimate – PE, and ultimate – U, syllable stress). The experimenter read the target word aloud and children were asked to say aloud the number 1, 2, or 3, corresponding to the first, second or third syllable, according to what syllable they perceived as the stressed one. Children had to correctly answer at least three consecutive items. Participants who did not reach this cut-off were excluded.

At the end of the selection process, 48 participants remained, and took part in the experiment: 18 TD children (mean age = 9.85, *SD* = 0.67, range 8.9–10.7; 10 males), 15 children with DD (mean age = 10.3, *SD* = 0.87, range 9.28–11.9; 5 males) and 15 normotypical adults (AC; mean age = 29.2, *SD* = 11.3, range 20.5–56.8; 6 males).

All participants were native Italian speakers, and all children were regularly attending school. All children’s parents/legal guardians and adult participants signed written informed consent. The study had been approved by the Ethics Committee of the University of Pavia, according to standards of the Helsinki Declaration (1964).

### Materials

#### Standardized Language and Cognitive Tests

Here we list all the tests that were used either as selection criteria for, or to characterize, the TD and DD groups. All these tests were standardized on the Italian school-age population.

The following tests were administered to TD children in order to evaluate their linguistic and general cognitive abilities.

(i) A test of morphosyntactic comprehension and production (CoSiMo – described in Cantiani et al., 2015). This unpublished test has been standardized in a large, well-controlled normative sample from various regions of Italy. Three subtests were administered: a direct to indirect speech transformation task (“speech”), an active to passive voice transformation task (“voice”) and a task on free morphology where the use of clitic pronouns has to be judged and corrected when necessary so as to render the same meaning as a target sentence (“clitics”). The battery relies on the implicit use of morphosyntactic transformations and avoids any reference to explicit rules, giving examples of transformations as instructions.
(ii) A test of sentence repetition – SR (Ferrari et al., 1981), requiring the participants to repeat a list of 14 sentences of increasing length and complexity. One point was assigned for each sentence repeated correctly after the first administration, 0.5 after the second administration.
(iii) Colored Progressive Matrices - CPM (Raven, 1947; Belacchi et al., 2008).
  Tests (i) and (ii) were administered also to DD children in order to describe their language abilities. The scores of the following tests were additionally retrieved from clinical records as selection criteria for the DD group:
(iv) WISC-IV (Orsini et al., 2012) for IQ assessment.
(v) The MT-2 text reading test (Prove di lettura MT-2 for primary and for secondary school, Cornoldi et al., 2011), a text-reading task meant to assess reading abilities for meaningful material. It provides separate scores for speed and accuracy. Texts increase in complexity with grade level; age norms are provided for each text.
(vi) A test of word and non-word reading (DDE-2, Sartori et al., 2007). This test assesses speed and accuracy (expressed in number of errors) in reading word lists (4 lists of 24 words) and non-word lists (3 lists of 16 non-words) and provides grade norms from the second to the last grade of junior high school.

Table 1 reports descriptive statistics for the listed tests in the TD and DD groups.

**Table 1:** Mean (±SD) standardized scores of the TD and DD groups.

	IQ	CoSiMo (morphosyntax)			SR (Sentence Repetition)	
		Speech	Voice	Clitics		
TD	103.9 ± 15.8	−0.38 ± 0.71	−0.53 ± 0.81	−0.26 ± 0.8	0.24 ± 0.64	
DD	104.6 ± 16.9	−0.91 ± 1.21	−1.02 ± 0.82	−0.13 ± 0.97	−0.77 ± 1.76	
	*t* = −0.13, *p* = 0.551	*z* = 1.159, *p* = 0.123	*t* = 1.682, *p* = 0.051	*t* = −0.417, *p* = 0.66	*z* = 1.556, *p* = 0.06	

	**MT-2 (text reading)**		**DDE-2 (word and non-word reading)**			
	**Speed**	**Accuracy**	**Words Speed**	**Words Accuracy**	**Non-words Speed**	**Non-words Accuracy**

DD	−1.48 ± 0.47	−1.88 ± 1.24	−3.54 ± 2.87	−3.03 ± 1.88	−1.98 ± 1.46	−2.29 ± 1.36

*IQ was obtained from the WISC-IV (Orsini et al., 2012) for the DD children, and from the CPM test (Raven, 1947; Belacchi et al., 2008) for the TD children. Z scores are reported for all other tests. Statistical tests are reported for the comparisons between groups (p-values are one-tailed in the direction of deficit in the DD group). Mann–Whitney tests were performed in cases of violation of homoscedasticity or normality assumptions (Z scores are reported in these cases).*

#### Experimental Stimuli

The stimuli of the experimental task were derived from three non-words /dididi/, /gugugu/, and /tatata/. The vowels /e/, /ε/, /o/, /ɔ/ were not used in order to avoid any biases that tend to be pronounced differently in different regions of Italy and to change their characteristics depending on stress position. Each non-word was recorded by the same native Italian speaker; specifically, several instances were produced and recorded, and the clearest and most recognizable recording (as judged by six adult listeners with an agreement of at least 4/6) was selected, so as to have one recording for each of the different stress patterns: AP, PE, and U, thus producing a set of nine basic recordings. Recordings were carried out with an entry-level dynamic microphone in a quiet environment, without changing distance from the device, and trying to keep a consistent loudness between the takes. Recordings were performed through the PRAAT software (Boersma and Weenink, 2018) and stored in WAV files as a single-channel Pulse Code Modulation stream, with a sampling frequency of 44100 Hz and a bit-depth of 16 bits.

Each of the nine sounds were analyzed to extract the three features that, according to the literature, differentiate stressed from non-stressed syllables:

• *Duration* of the vowel of the syllable. The pauses between syllables were not considered/manipulated, as their dilation or contraction would have altered the perception of the corresponding occlusive consonant;
• *Intensity* or loudness: the root mean square (RMS) of the whole syllable. We chose this feature since the instantaneous peak contour would not be relevant, as it mainly depends on the phase of single harmonics. Moreover, shorter RMS windows would lack in significance as a measure of loudness.
• *Pitch*, a shortcut to refer to the pitch *contour* across the duration of the syllable. We matched the full pitch contour and not just the average pitch in order to take into account differences between, e.g., rising or falling pitch patterns.

**FIGURE 1:**
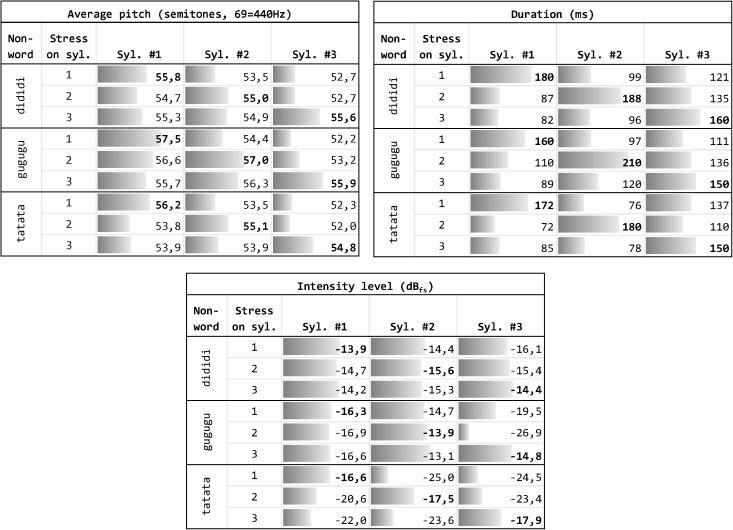
Measurement of the basic features of the nine original recordings. Values concerning the stressed syllable are reported in bold.

Figure 1 illustrates the different features of each original audio file.

The nine original files were then manipulated (by means of a dedicated software, Steinberg Cubase) in order to obtain 72 new stimuli, in which the duration, intensity and pitch stress patterns were dissociated and varied independently. For instance, there were stimuli with the duration profile of an AP stimulus, the intensity profile of an U stimulus, and the pitch profile of a PE stimulus, others with U duration and pitch patterns but an AP intensity pattern, and so on. All possible combinations were generated, for an overall set of 81 stimuli (3 × 3 × 3 × 3, i.e., duration profile, AP/PE/U, by intensity profile, AP/PE/U, by pitch profile, AP/PE/U, by non-word, /dididi/, /gugugu/, /tatata/ – the nine original stimuli belong to this overall set). This design allowed us to disentangle the contribution of each parameter to the perceived stress position from the contributions of the others.

Thanks to the VariAudio and Free Warp functionalities, all of the previously mentioned manipulated audio files sounded very natural. We explicitly avoided producing artificial-sounding stimuli, as these might have biased the results (by giving the listener some hints as to the manipulation, with unpredictable effects on performance). Albeit synthetic in origin, our natural-sounding material was likely to be processed by the listener in the same way as real-world stimuli.

The exact software procedure applied to produce the stimuli is detailed in the Supplementary Materials.

### Procedure

All participants were individually tested in a quiet room, seated next to the experimenter, in front of the 11.6″ screen of an Acer Aspire V5-131 × 64 laptop computer. They listened to the stimuli through AKG K518DJ headphones. One of three pre-randomized lists of items were randomly assigned to each participant (the algorithms were designed using Psychopy software: Peirce, 2007). A visual stimulus, showing the target non-word written in capital letters and without diacritical stress, e.g., TATATA, appeared on the screen simultaneously with the audio stimulus. Participants had to judge the stress position, by pressing the keys “1”, “2,” or “3” (corresponding to AP, PE, U) with the index finger of their dominant hand. No feedback was given regarding response accuracy. The written non-word remained on the screen until the participant pressed one of the three keys; 1 second later the next trial began. No time limits were given for responding, but stimuli could not be played twice.

### Statistical Analyses

#### Consistency Scores

A number of ‘Consistency scores’ were obtained from the performance of each participant and analyzed. A Consistency score expressed to what degree the participant’s responses matched a given parameter of the stimulus (duration, intensity, or pitch). Taking the DC as an example and supposing that on a given trial the duration pattern was that of an AP stress, the participant’s responses were scored as follows: an AP response, consistent with the Duration pattern, was granted a score of 1; any other response was given a score of 0. If the Duration pattern was Penultimate (PE), 1 was granted to a PE response, and 0 otherwise; if the Duration pattern was Ultimate (U), 1 was granted to an U response, and 0 otherwise. This procedure was repeated all across the trials, obtaining a list of binary 0-1 scores; the proportion of 1 scores expressed the responses’ degree of consistency with Duration. This proportion ranges from 1/3 (chance level), that is, the expected proportion of Duration-consistent responses if Duration has no influence on responses, and 1 – the ideal case where Duration directly determines the response on every trial. To have a measure with more transparent meaning, we rescaled the score to bring its range from [0,1] with 1/3 chance level, to [−0.5,1] with 0 chance level (this is achieved by applying a 1.5*x* – 0.5 transformation to the *x* original value). Thus the DC is expected to be zero if Duration is not considered at all in the judgements, to be 1 when it determines all responses, and to assume intermediate values when its influence on responses is partial. A negative score indicates that some process led to choose the ‘correct’ response according to Duration less often than chance would predict. For instance, if a participant tends to perceive or classify AP Duration stress patterns as PE, the Duration scores will come out negative. The extreme value, −0.5, corresponds to the (purely theoretical) case in which the ‘correct’ response according to Duration is never given.

By applying the same procedure to Intensity and Pitch, we ended up having three Consistency scores: Duration Consistency (DC), Intensity Consistency (IC), and Pitch Consistency (PC). Clearly, the three scores constrain one another, because one cannot be fully consistent (score = 1) with more than one criterion. Thus, while a participant whose responses fully reflect Duration will have (DC, IC, PC) = (1, 0, 0), a participant in whom Duration ‘wins’ in half the trials and Intensity ‘wins’ in the other half, will obtain (0.5, 0.5, 0). The sum of the three scores, DC + IC + PC (which usually does not exceed 1) expresses the Overall Consistency (OC) of responses with any criterion. Thus for instance, a participant whose responses are consistent with Duration in ¼ of the trials, with Intensity in another ¼, and given at random in the remaining ½ the trials, obtains (0.25, 0.25, 0), with OC being 0.25 + 0.25 + 0 = 0.5, correctly reflecting the fact that the participant considered some criterion to generate his response in half the trials. Clearly, if responses are totally unrelated to the three criteria and given at random, OC turns out to be 0. This does not necessarily mean that the participant selects the AP, PE, and U responses in equal proportions: thanks to the fully balanced design, any response bias – any preference AP, PE, or U responses cancels out and provides no spurious contribution to the Consistency scores. All such features of the present measures were confirmed by means of simple Monte Carlo simulations.

As a further step, nine specific Consistency scores were derived for each combination of Parameter (Duration, Intensity, Pitch) and Stimulus Stress Pattern (AP, PE, U). This allowed us to understand whether the effect of a given parameter varies according to the position of the stress pattern (e.g., Duration might have a greater impact on responses in the AP than in the PE and U configurations, etc.).

#### Statistical Tools

General Linear Model (GLM) was used to analyze Consistency scores within groups. This proved adequate insofar as the histograms of the residuals obtained from the most complex GLM model did not show important departures from normality. By contrast, non-parametrics were used to compare different Groups as their scores could show important departures from normality. Because of these violations, the GLM interactions between Group and some within-participant variable(s), were little reliable and were either interpreted with caution or omitted from the present discussion.

On a first step, we determined whether the three groups, adults, TD and DD, differed in their overall ability to carry out the task. On a second step, we explored the relative use of the three parameters, Duration, Intensity and Pitch, in each of the three groups. On a third step, we explored whether each parameter had differential effects according to Stress Pattern.

Additionally, we carried out a set of GLM and Partial Correlation analyses to further explore the role of some predictors – age, morphosyntactic comprehension/production, sentence repetition, and reading parameters, on the Consistency scores.

Effect sizes were reported in terms of partial eta-squared (η^2^) for GLM analyses. Greenhouse–Geisser correction was applied to three-way within-subjects effects (hence the non-integer degrees of freedom).

No correction for multiple comparisons was applied, being the analyses planned comparisons with explicit and clear-cut expectations. As to the impact of various predictors on Stress Perception parameters, for which we had no clear expectations, no correction was applied either, due to the presence of high mutual correlations between the variables. Given the novelty of both the stimuli used and the Consistency scores derived from them, we had no reliable way to estimate the effect sizes before the experiment. Hence we could not perform a power analysis, which is an acknowledged limitation of the present study.

## Results

### Overall Consistency Score

**FIGURE 2:**
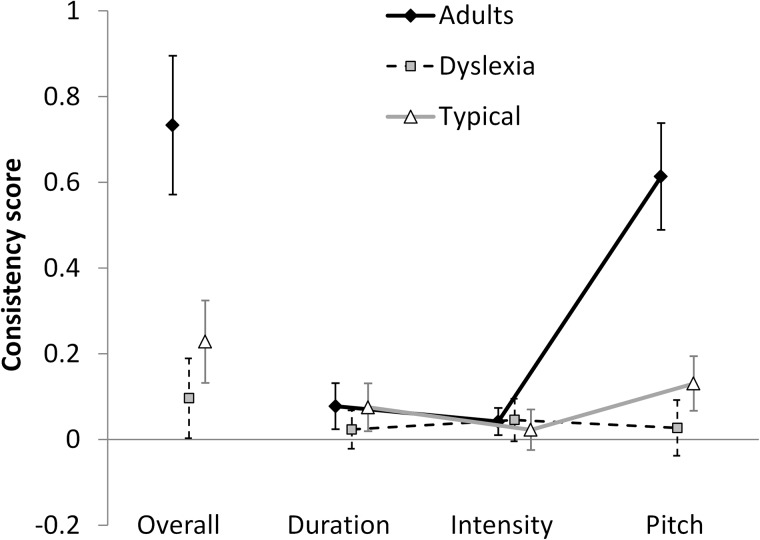
Overall Consistency scores and specific Consistency scores (for Duration, Intensity, and Pitch) of Adults, Typical Developing children and Dyslexic children. Each Overall score is the sum of the three specific scores. The Consistency scale (vertical axis) extends from –0.5 (perfect in-consistency) to 0 (chance level) to 1 (perfect consistency). Error bars report 95% confidence intervals.

When looking at the general ability to solve the task, as measured by the OC, all three groups obtained an above-chance performance, albeit DD children barely surpassed this threshold [Wilcoxon tests: DD children, *z* = 2.019, one-tailed *p* = 0.022; adults: *z* = 3.411, one-tailed *p* = 0.001; TD children: *z* = 3.436, one-tailed *p* = 0.001]. However, there were massive differences between groups [Kruskal–Wallis, χ^2^(2, *N* = 48) = 23.235, *p* < 0.001]. The left side of Figure 2 shows the pattern. As expected, adults performed much better than TD children: the average OC score of the former, 0.733, was more than three times larger than that of the latter, 0.228, [Mann–Whitney, *z* = 3.926, one-tailed *p* < 0.001]; in turn, TD children slightly outperformed dyslexic children, whose OC score was 0.096 [*z* = 1.701, one-tailed *p* = 0.044]. Thus, recalling that OC = 1 corresponds to a perfectly consistent performance (according to any criterion) and that OC = 0 corresponds to random responses, adults (0.733) were reasonably close to an optimal performance, while children of both groups were very far from it (0.228 and 0.096).

**FIGURE 3:**
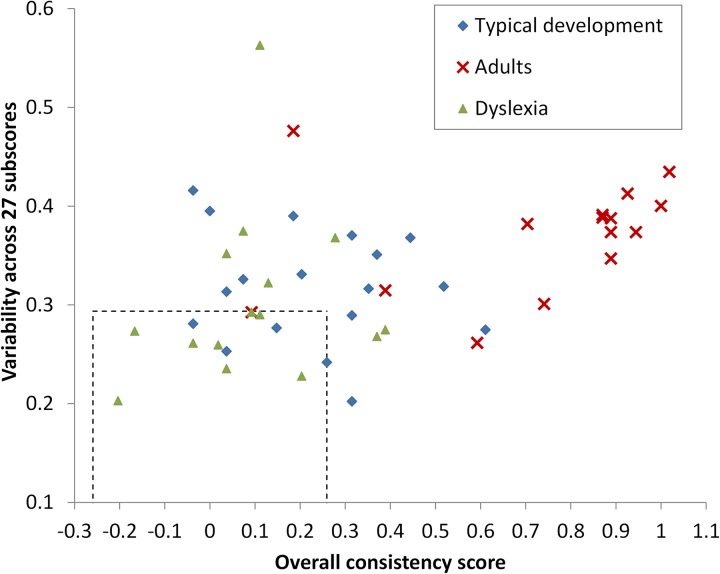
Overall Consistency scores are plotted against performance variability, which was estimated as the standard deviation across 27 different subscores. Each dot corresponds to a participant. The dashed-outlined box shows the region where Monte Carlo-generated random performances fall with 95% probability on each axis.

While, as shown above, performance was *on the average* above chance in all three groups, a significant proportion of participants had a performance level compatible with random selection of responses, and this proportion was largely different in different groups. A Monte-Carlo simulation study (*N* = 10,000) showed that a reasonable (95%) random-response range for OC is between −0.259 and +0.259. Two out of 15 adults (13%) had a score in the random range, while 9/18 (50%) TD children, and 12/15 (80%!) of DD children did so. This picture, however, has the limitation that there might, in principle, be participants who fell in the ‘random’ range, with an OC relatively close to zero, not because they responded randomly, but because they had opposite (positive and negative) consistencies canceling out each other (e.g., using Pitch in the ‘correct’ way, but using Duration in an unexpected way, e.g., systematically selecting the PE response when the Duration pattern is AP). To rule out this criticism, we also studied the inherent variation of the OC score. Indeed OC is actually the mean consistency across 27 atomic subscores (those identified by the 3 × 3 × 3 Non-word × Stress Position × Parameter design); the Monte Carlo study taught us that if participants had been selecting responses at random, the standard deviation (SD) of the 27 consistency values would have been (95%) below 0.294. Figure 3 plots the OC values against the SD values for all participants, and the random-response range is shown as a box with dashed borders. In this perspective, the picture is different: 1/15 adults (7%), 3/18 TD children (17%), and 8/15 DD children (53%) fell in the random-response region.

Overall, while DD and TD children are much closer to random performance than AC, there are hints that some non-random behaviors characterize all groups.

### Main Effects of Parameter

As a second step, we studied to what degree each parameter, Duration, Intensity or Pitch, influenced responses. To do so, we looked at the variable Parameter (DC, IC, PC) within each group. The right side of Figure 2 shows the patterns.

#### Adults

Adults showed a very large effect of Parameter [*F*(1.304,18.261) = 88.178, *p* < 0.001, η^2^ = 0.863]. Pitch proved to be by far the most influential factor in determining perceived stress pattern in this group, with a Consistency score of 0.614. Duration and Intensity were much less effective, with 0.078 and 0.042 Consistency scores. However it is important to note that the latter contributions were both significantly above chance [Wilcoxon: *z* = 2.737, one-tailed *p* = 0.003, and *z* = 2.367, one-tailed *p* = 0.009, respectively]. Pairwise comparisons confirmed the huge advantage of Pitch over Duration [*F*(1,14) = 93.763, *p* < 0.001, η^2^ = 0.87] and over Intensity [*F*(1,14) = 99.554, *p* < 0.001, η^2^ = 0.877]. Duration and Intensity influenced responses to a similar degree [*F*(1,14) = 2.029, *p* = 0.176, η^2^ = 0.127].

#### Typically Developing Children

TD children also showed an effect of Parameter [*F*(1.998, 33.967) = 4.108, *p* = 0.025, η^2^ = 0.195]. Pitch contributed with a Consistency score of 0.131, Duration of 0.075 and Intensity of 0.023. Only Pitch and Duration contributed significantly above chance [Wilcoxon: *z* = 3.198, one-tailed *p* < 0.001, and *z* = 2.669, one-tailed *p* = 0.004, respectively], while Intensity failed to reach this threshold [*z* = 0.83, one-tailed *p* = 0.203]. Pairwise comparisons between Parameters showed that only the difference between Intensity and Pitch reached significance [*F*(1,17) = 8.462, *p* = 0.01, η^2^ = 0.332], while the Pitch-Duration [*F*(1,17) = 2.122, *p* = 0.163 η^2^ = 0.111] and the Intensity-Duration comparisons [*F*(1,17) = 1.927, *p* = 0.183, η^2^ = 0.102] failed to do so.

#### Dyslexic Children

DD children did not show an effect of Parameter [*F*(1.617,22.634) = 0.224, *p* = 0.754, η^2^ = 0.016]. So there is no evidence that the (slightly) above-chance performance by this group depends on some specific parameter.

#### Overview

Figure 2 clearly depicts this general pattern of results. Adults and TD children seem to show a *qualitatively* similar pattern – Duration and Intensity are used to a small degree^1^, and Pitch is used to a higher degree. As for Pitch, a *quantitative* difference emerges, in that its influence is much higher in adults. By contrast, DD children seem to show a qualitatively different pattern^2^: albeit they perform slightly above chance, there is no hint as to what (average) combination of Parameters are being used by them – their average pattern seems flat.

### The Effects of Parameters in Different Stress Positions

As a last step, we explored whether the Consistency scores of the various Parameters were modulated by Stress Position. Given the large overall-performance differences between groups, these analyses were again carried out on each group separately.

#### Adults

**FIGURE 4:**
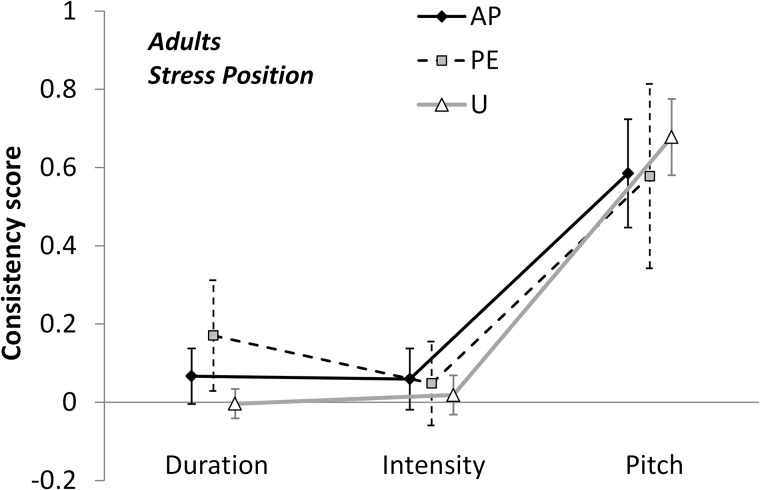
Consistency scores by Adults are plotted against Parameter, as a function of Stress Position (AP, antepenultimate; PE, penultimate; or U, ultimate). Error bars are 95% confidence intervals.

Adults showed a significant effect of Parameter [*F*(1.304,18.261) = 88.178, *p* < 0.001, η^2^ = 0.863] and of Parameter × Stress Position [*F*(2.901,40.609) = 9.278, *p* < 0.001, η^2^ = 0.399]. The inspection of the plot (Figure 4) clarifies the meaning of such an interaction. While Pitch and Intensity influenced responses to a similar degree across the three Stress positions [Pitch: *F*(1.595,22.33) = 0.758, *p* = 0.452, η^2^ = 0.051; Intensity: *F*(1.535,21.489) = 0.238, *p* = 0.732, η^2^ = 0.017], Duration seemed to be most effective in PE position [*F*(1.409,19.733) = 3.961, *p* = 0.048, η^2^ = 0.221].

#### Typically Developing Children

TD children also showed a significant Parameter × Stress Position interaction [*F*(2.982,50.698) = 4.311, *p* = 0.009, η^2^ = 0.202].

**FIGURE 5:**
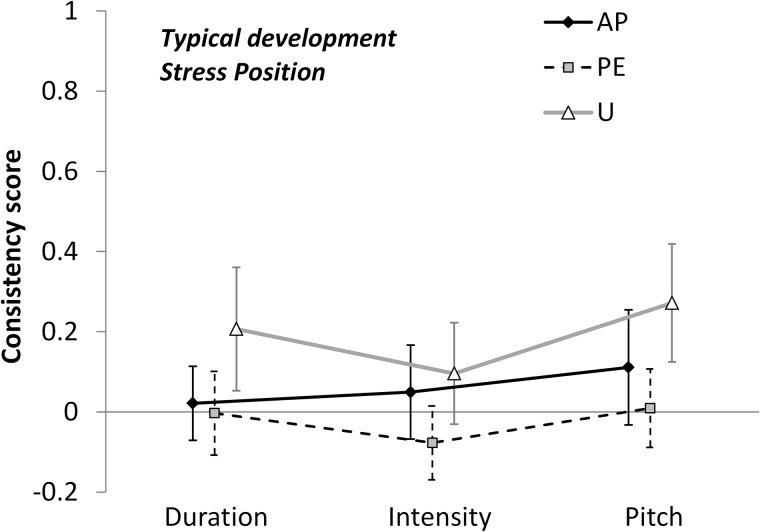
Consistency scores by Typically Developing children are plotted against Parameter, as a function of Stress Position (AP, antepenultimate; PE, penultimate; or U, ultimate). Error bars are 95% confidence intervals.

Stress Position modulated the effect of Parameters as follows (Figure 5). The effect of Pitch was largest in U, intermediate in AP, and smallest in PE, for which the effect was at chance level [*F*(1.815,30.852) = 3.936, *p* = 0.034, η^2^ = 0.188]. By contrast, Duration seemed to be mostly affecting U stress patterns, while being at chance level for PE and AP [*F*(1.731,29.431) = 3.464, *p* = 0.051, η^2^ = 0.169]. No Stress-Position effect was found for Intensity [*F*(1.946,33.079) = 2.261, *p* = 0.121, η^2^ = 0.117].

#### Dyslexic Children

DD children showed a marginal Parameter x Stress Position modulation [*F*(2.973,41.622) = 2.93, *p* = 0.045, η^2^ = 0.173]. However, none of the within-Parameter analyses revealed any significant effect.

### Other Predictors

The question then arises whether this pattern of results is modulated by some predictors that were available from our TD and DD samples. Namely, we wondered whether age, morphosyntactic abilities and especially, reading abilities (which were measured in the DD group), have an impact on performance on the stress perception task. Being aware of the lack of power of an analysis including all predictors at once, we explored the dataset in a stepwise fashion, by including only variables that proved significant on a previous step, and if OC was not affected by some predictor, its specific effects on the Duration, Intensity and Pitch components were not studied.

#### TD and DD Children: Effects of Age and Linguistic Abilities

Age, Morphosyntactic abilities (the sum of the three subscores of the CoSiMo battery), and Sentence Repetition were available for both TD and DD children, so the effects of these predictors were analyzed by GLM to partial out possible group differences (Table 2).

**Table 2:** Impact of a set of predictors on Stress Perception parameters.

	Overall Consistency Score	Duration	Intensity	Pitch
Age	*F*(1,29) = 0, *p* = 0.495, η^2^ = 0			
Age × Group	*F*(1,29) = 1.904, *p* = 0.178, η^2^ = 0.062			
CoSiMo	***F*(1,29) = 5.723, *p* = 0.012, η^2^ = 0.165**	***F*(1,29) = 4.772, *p* = 0.019, η^2^ = 0.141**	*F*(1,29) = 0.028, *p* = 0.434, η^2^ = 0.001	***F*(1,29) = 3.139, *p* = 0.043, η^2^ = 0.098**
CoSiMo × Group	*F*(1,29) = 0.805, *p* = 0.377, η^2^ = 0.027	*F*(1,29) = 0.565, *p* = 0.458, η^2^ = 0.019	*F*(1,29) = 1.391, *p* = 0.248, η^2^ = 0.046	*F*(1,29) = 0.059, *p* = 0.809, η^2^ = 0.002
SR	*F*(1,28) = 0.009, *p* = 0.463, η^2^ = 0			
SR × Group	*F*(1,28) = 0.513, *p* = 0.48, η^2^ = 0.018			

*CoSiMo, Morphosyntax. SR, Sentence Repetition. P-values for main effects are one-tailed in the expected direction (the younger, the worse the performance; the worse the performance on the predictor, the worse the performance on stress perception); p-values from interactions are two-tailed. Significant effects are reported in bold. Effect sizes are reported as Eta-squared (η^2^).*

Age and Age × Group (where Group is TD vs. DD children) were studied at a first step, and proved non-significant as predictors of the OC score of the stress task. Hence, at least in the short age range that we explored (8.95–11.87 years), age does not account for the tendency by some DD children to express random responses, which corresponds to an OC score close to zero.

On a second step, the morphosyntactic ability score (sum of CoSiMo subtests) was used as predictor of OC (Age was used as a covariate, which is equivalent to using age-standardized CoSiMo scores, but again it proved non-significant, so it was removed from the analysis). As shown in Table 2, CoSiMo significantly affected performance on the stress perception task in the expected direction: the better the morphosyntactic abilities, the better the OC score. More in detail, the Duration and Pitch components contributed to such an effect – i.e., those components that were found to be relevant in the perception of stress position by TD children.

On a third step, Sentence Repetition was studied as a predictor (Age and CoSiMo scores were used as covariates, and only CoSiMo confirmed to have a reliable impact), however, this failed to significantly predict the OC score.

#### DD Children: Effects of Reading Scores

The relationship between OC scores and reading was tested in the DD group. We focused on the word and non-word reading tests (DDE-2, Sartori et al., 2007) because these rely on identical material across the tested ages. Both reading accuracy and reading speed (seconds per syllable, see Toraldo and Lorusso, 2012, for theoretical justification) were analyzed. Partial correlations of such scores with stress perception parameters are reported in Table 3.

**Table 3:** Impact of reading performance (DDE-2, Sartori et al., 2007) on Stress Perception parameters in the DD group.

		Overall Consistency score	Duration	Intensity	Pitch
Words	Accuracy	***r* = 0.594, *p* = 0.013**	*r* = 0.196, *p* = 0.251	*r* = −0.133, *p* = 0.675	***r* = 0.666, *p* = 0.005**
	Speed (sec/syll)	***r*** = **-0.788, *p* < 0.001**	***r*** = **-0.734, *p* = 0.001**	*r* = −0.232, *p* = 0.203	***r*** = −**0.447, *p* = 0.047**
Non-words	Accuracy	***r* = 0.544, *p* = 0.027**	*r* = 0.089, *p* = 0.386	*r* = −0.351, *p* = 0.88	***r* = 0.803, *p* < 0.001**
	Speed (sec/syll)	***r*** = **-0.636, *p* = 0.007**	*r* = −0.415, *p* = 0.07	*r* = −0.175, *p* = 0.275	*r* = −0.417, *p* = 0.069

*Partial correlations are reported controlling for the contributions by Age and CoSiMo scores (when significant). All p-values are one-tailed in the expected direction (the worse the reading performance, the worse the performance on stress perception). Significant correlations are reported in bold.*

**FIGURE 6:**
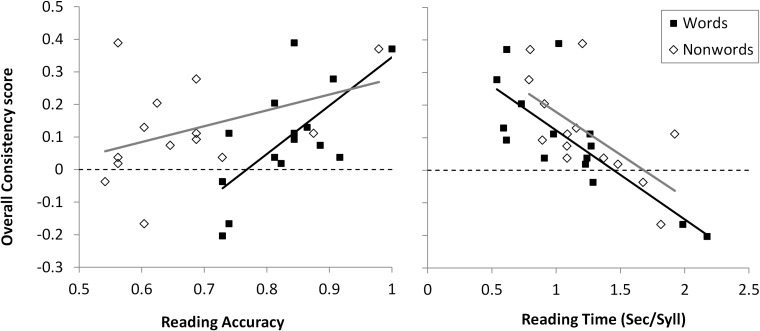
Overall Consistency scores on the Stress Perception task (vertical axes) are plotted against Reading Accuracy (proportion correct, **left panel**) and Reading Time (seconds per syllable, **right panel**) on the DDE task, for children with Developmental Dyslexia. Dashed horizontal lines report chance level (OC = 0); solid black lines are the regressions for words (filled squares); solid gray lines are the regressions for non-words (open diamonds).

All four reading scores were rather robust predictors of OC; in most cases (see Table 3) Pitch was the component that was best predicted by reading performance. Figure 6 shows the predictive pattern, which is rather tight, with (partial) correlations between stress perception and reading performance ranging from 0.544 to 0.788 in absolute value.

## Discussion

Systematic manipulation of the pitch, duration and intensity profiles of three Italian trisyllabic non-words produced a series of 81 stimuli. These stimuli were judged with respect to stress position (perceived on the ultimate, penultimate or antepenultimate syllable) by three groups of participants: children with dyslexia, TD matched on age and gender, and normotypical adults.

We had a number of predictions based on the previous literature which we will now discuss in turn.

### The Dominance of Pitch Over Duration

A first prediction, based on previous literature, was that *duration* should have been the critical parameter in determining stress assignment while processing Italian non-words. This hypothesis was contradicted by our results, which showed that the *pitch* component is the most reliable acoustic cue in stress perception for both adults, in whom this dominance is very strong, and TD children, who showed a similar but quantitatively less marked pattern. Although many studies on Italian stress perception underlined the role of duration (e.g., Bertinetto, 1980), other studies have shown that pitch plays an important role in stress perception in many languages (e.g., Fant et al., 1991; Hasegawa and Hata, 1992; Antoniou et al., 2015). Moreover, the discrepancy between the results of the present work and those of previous studies on Italian lexical stress assignment may be due to differences in the stimuli: indeed to produce them, we used a software (Steinberg Cubase 5) which is more sophisticated than those typically employed in the literature. Most importantly, however, we used non-words controlled for semantic and phonological neighbors and for coarticulation effects, while words and pseudo-words are typically used in the literature. In essence, the present study shows the “barebones” of the machinery of stress assignment, in a particularly pure condition where there can be no plausible influence by lexical processing. Stimuli were natural syllables, but the same syllable was repeated three times across the string, thus producing a stimulus that is neutral at both the semantic and the lexical levels of analysis. Thus we may hypothesize that the reason why previous authors found that duration, and not pitch, was the critical feature in stress perception, is that some interaction occurs between the lexical/semantic levels and the early acoustic analyses in this process (Arciuli and Colombo, 2016), which changes the relative weight of the parameters in determining performance. Note that even in studies using pseudowords there might have been an implicit lexical contribution, as pseudowords partially activate the phonological lexicon and do so as a function of orthographic/phonological similarity (Rosson, 1983) (e.g., the pseudoword /tavoga/ is very likely to activate the lexical node /tavola/, table). Also, differently from experiments using words and pseudowords, across our experiment participants listened to the same strings, /tatata/ /gugugu/, /dididi/, over and over, which likely contributed to a further swamping of any, however, small, lexical activation. Overall, further research is needed to investigate the possible top–down effects of complete or partial lexical access on the acoustic processing that eventually leads to stress perception.

Another source of insight as to the role of pitch can be found when comparing the present results to those by Antoniou et al. (2015). These authors found that pitch perception was a stronger predictor of language ability in Chinese as compared to rhythm perception (which failed to have any impact at all on performance in their tasks); they suggested that the acoustic parameters predicting language development are language-specific, and that tone languages such as Chinese have different predicting patterns as compared to Western languages. Momentarily neglecting the many differences between Antoniou et al.’s (2015) and the present work, both in the experimental tasks and in the dependent variables (language tasks versus reading), we would (if anything) have predicted that Italian participants would behave more similarly to English or other European speakers/readers than to Chinese speakers. By contrast, pitch turned out, both in ours and in Antoniou et al.’s (2015) study, to be the most relevant parameter determining stress processing and perception. Even if Italian is not a tone-based language, it partially differs from most other European languages, especially Germanic ones, in the very range of pitch variations produced by its speakers (Hirst and Di Cristo, 1998). Even more precisely, while tone determines lexical identity in Chinese, pitch variations in Italian are the vehicle for prosody-based pragmatic communication, conveying emotion and meaning (e.g., questioning and statement: Hirst and Di Cristo, 1998; D’Imperio, 2002). This may suggest that Italian speakers are more used to process pitch variations and therefore their ability in processing pitch is higher than for speakers of other languages. Indeed, since the variation in syllable duration is limited in Italian (Nespor et al., 2011), it is reasonable to hypothesize that speakers and listeners of this language base their production/perception of lexical stress on other parameters. Furthermore, if pitch is processed in order to extract pragmatic cues, this might explain the impressive growth in sensitivity that we observed for this parameter across the lifespan: pragmatics is doubtlessly the linguistic component which develops more slowly and more gradually during life, along with experience in interaction with other people across different contexts and conditions.

### Typically Developing Children vs. Adults

The second prediction derived from the literature was that TD children and adults should have shown similar performances when perceiving acoustic parameters. This hypothesis was also falsified. Indeed, sensitivity to pitch turned out to be lower in children than in adults, although pitch was found to be the most relevant parameter also for TD children. To our knowledge, no previous study investigated the development of acoustic parameters processing involved in stress assignment. Moreover, there are studies on pitch perception in infants but they mostly used musical rather than speech stimuli (Trehub, 2001; Plantinga and Trainor, 2005) or they focused on pitch characteristics of infant-direct speech and its influence on infants’ discrimination ability (Marcos, 1987; Trainor and Desjardins, 2002).

### Dyslexic vs. Typically Developing Children

A final prediction was that children with DD should have been less sensitive than TD peers to changes in the acoustic parameters while processing stress position. This hypothesis was confirmed. Indeed, our DD children did not seem to rely on any parameter in their judgments, and rather gave random responses, which point to a general inability to process the various acoustic modulations that normally contribute to stress perception. Thus, in line with Goswami et al. (2013), our DD children showed an impaired sensitivity to syllable stress compared to their TD peers (and adults).

Interestingly, performance on lexical stress perception was found to correlate with morphosyntactic abilities in the sample of children (including TD and DD), and with reading abilities in the group with DD. Such correlations support the idea that perception of stress helps building more stable and well-defined phonological and orthographic representations of the words that will be thus more easily retrieved during reading (Elbro and Jensen, 2005; Perfetti, 2007). Even more crucially, these correlations highlight the strict connections existing between prosodic skills and written text decoding, as well as between prosody and other language abilities. Indeed, a relationship is often described between reading and phonological abilities at the phonemic level (e.g., Bogliotti et al., 2008; Hämäläinen et al., 2009, 2013), possibly extending to the syllabic or onset/rime level (e.g., Goswami et al., 2010, 2013; Frey et al., 2018), but more rarely encompassing lexical prosody for multisyllabic words. An undifferentiated approach to prosody though, not distinguishing between the various levels, can fail to capture the very specific and articulated links between functions both within and across linguistic domains (see Leong and Goswami, 2014). Furthermore, single and specific aspects of prosody seem to be involved in different, specific learning abilities: reading versus writing, speed versus accuracy, word versus non-word versus meaningful text reading, etc. In the context of the Letter-Speech Sound Integration issue, it has been proposed (Zhang and McBride-Chang, 2010) that auditory sensitivity impacts speech perception, with temporal processing especially influencing the segmental/phonemic level while rhythmic processing would more specifically affect the suprasegmental/prosodic level. In turn, segmental and suprasegmental processing would influence literacy acquisition through phonological processing on the one hand, and morphological awareness on the other hand. Nonetheless, in the model proposed by these authors, sensitivity to speech prosody such as stress may also influence speech perception at the segmental level, by facilitating spoken word recognition and enhancing the perception of phonemes according to the Lexical Restructuring Hypothesis (Metsala and Walley, 1998; Wood et al., 2009). In this perspective, sensitivity to speech rhythm could explain individual differences in reading ability beyond, and independently of, the contribution of phonological awareness.

This also suggests that the efforts to train pre-school and early school children in phonemic awareness tasks could be even more effective in preventing or remediating reading failure if complemented by exercises requiring to perform prosodic analysis at various levels (as shown also by Thomson et al., 2013) or by emphasizing the rhythmic structure of the linguistic stimuli (e.g., Bonacina et al., 2015). Moreover, better stress perception could contribute to the development of syntactic skills (in a bidirectional manner) both by providing clearer representations of lexical entries and by helping disentangle ambiguous syntactic structures through prosodic cues (e.g., Frazier et al., 2006; Snedeker and Yuan, 2008; Caccia and Lorusso, 2019). With regard to writing skills, Angelelli et al. (2010) showed that Italian children with DD and, more generally, with specific learning disorders, tend to omit the (compulsory) diacritic marks when writing ultimate-stressed words. The present study suggests that such difficulties possibly lie in stress perception rather than in orthographic stress representation. Specifically, the DD children of our study seemed to have lacking awareness of lexical stress position, suggesting that orthographic difficulties actually originate at the metaphonological level. In the light of this result, it could be interesting to investigate whether this metaphonological deficit, in turn, arises at a low level of acoustic analysis or at a higher level of integration in an abstract stress-position representation. To this purpose, ERPs might be recorded in future studies during the listening of “swapped” acoustic parameters stimuli, thus allowing one to disentangle lower from higher components of stress perception. Moreover, ERP studies could be conducted also with younger children to define developmental trajectories and to identify possible early markers of language disorders.

In the light of the above, our results suggest that DD children show defective processing skills of acoustic parameters responsible for lexical stress assignment and therefore, their orthographic difficulties with diacritical markers should be supported and rehabilitated on the basis of strategies that are not based on acoustic analysis. Since the application of explicit grammatical rules are often challenging for children with DD (Pavlidou et al., 2009), more effective rehabilitation strategies should rely on (e.g.) visual memorization and recognition of grammatical morphemes (e.g., /-ò/, /-à/, as verb suffixes with diacritical marks) or frequent suffixes for nouns (e.g., /-tà/, from Latin “-tas”, in “felicitas” – “felicità”) or exception words (e.g., città, rondò, perché, così, etc.). Some intervention programs, such as the ones based on stimulation of hemisphere-specific strategies according to the balance model of dyslexia (see Bakker, 2006), rely on such strategies (Lorusso et al., 2006, 2011).

## Conclusion

In conclusion, the present study shows that pitch plays a crucial role in Italian stress perception, differently, for example, from stress perception in Spanish and Finnish which is characterized in terms of duration (Alfano et al., 2007; Eriksson et al., 2016). These findings seem to go in the same direction of a language-specific approach; indeed, following the LSAC hypothesis (Antoniou et al., 2015) the set of acoustic parameters required for the development of lexical stress perception (and possibly, of other aspects of language development) is language specific rather than universal, as postulated by the RDH (Goswami et al., 2011). This means that for languages that extensively use a specific acoustic cue (pitch, duration etc.), such acoustic parameter would be more important than the others and consequently would play a crucial role both in language processing and development (see also McBride-Chang et al., 2008). A cross-linguistic study with the same experimental paradigm would be useful to shed light on the role of acoustic parameters in determining lexical stress.

Beyond the role of single parameters for stress perception and language-specific differences, the present results confirm the role of prosody in reading and language development. More precisely, they highlight the need to extend the analysis of phonological abilities from a purely segmental to a broadly defined suprasegmental level to be able to detect and consider the subtle and likely bidirectional relationships linking low-level, perceptual abilities to the development of more and more complex oral and written language skills.

### Limitations

One limitation of the present study is that, given its relatively small sample sizes, statistical power is likely to be low. Given that we used dedicated stimulus types which (to our knowledge) were never used before, and also Consistency scores which were mathematically developed for the purpose, we could not run a reliable power analysis before the experiment. However, the main effects emerging from the analyses, which were the object of our theoretical discussion, are very large (e.g., the dominance of Pitch over the other parameters, or the differences in Consistency scores between adults and children), so that power limitations are unlikely to be an issue at least in those cases.

## Ethics Statement

All children’s parents signed informed consent. The study had been approved by the Ethics Committee of the University of Pavia, according to the standards of the Helsinki Declaration (1964).

## Author Contributions

MC contributed to the conception of the study, definition of the experimental design, organized and supervised data collection, and contributed to the writing of the manuscript. GP prepared the experimental stimuli and wrote a section of the manuscript. AT carried out all statistical analyses and took care of the interpretation and description of the results. AR administered the tests and coded them following the experimental protocol. LL participated in the definition of the experimental protocol. AO organized and supervised the recruitment of participants. ML contributed to the conception and definition of the experimental design, participated in the interpretation of results, contributed to the writing of the manuscript, and supervised the whole study. All authors contributed to manuscript revision, and read and approved the submitted version.

## Conflict of Interest Statement

The authors declare that the research was conducted in the absence of any commercial or financial relationships that could be construed as a potential conflict of interest.

## Footnotes

1 This is confirmed by the non-significant Group × Parameter interaction when only looking at Duration/Intensity and adults/TD children [*F*(1,31) = 0.124, *p* = 0.727, η^2^ = 0.004]. From the same analysis, the Duration/Intensity comparison fell short of significance [*F*(1,31) = 3.469, *p* = 0.072, η^2^ = 0.101]. However, because of the marked asymmetry in the distribution of adults’ scores, interactions are not completely reliable.

2 The Group × Parameter interaction, when comparing TD to DD children, gave *F*(1.947,60.348) = 2.932, *p* = 0.061, η^2^ = 0.086.

## Abbreviations

AP: PE, U, respectively, stress located on the AntePenultimate, Penultimate, Ultimate syllable
DAW: Digital Audio Workstation
DC: Duration Consistency score
DD: children with developmental dyslexia
IC: Intensity Consistency score
LSAC: language-specific auditory cue hypothesis
OC: Overall Consistency score
PC: Pitch Consistency score
RDH: rhythm detection hypothesis
TD: typically developing children.
