# Supplementary material for: Pitch as the Main Determiner of Italian Lexical Stress Perception Across the Lifespan: Evidence From Typical Development and Dyslexia
Source: Front Psychol. 2019 Jun 26;10:1458. doi: 10.3389/fpsyg.2019.01458 (PMC6611421; doi:10.3389/fpsyg.2019.01458)
Supplement: Supplementary file 1 [file Data_Sheet_1.pdf]

Supplementary Materials

Description of software procedure for stimulus production

All of the original nine recordings were loaded into Steinberg Cubase 5 (http://www.steinberg.net), a Digital Audio Workstation (DAW) capable of performing time dilation and contraction of sound files without affecting both timbre quality and r pitch, thanks to proprietary technologies called VariAudio and Free Warp. Furthermore, as many DAWs can do, Cubase lets the user sculpt the audio loudness envelope by means of an automation of the volume parameter. Changes in volume can be made according to the level meters included in the DAW, a key feature necessary to obtain precise adjustments.

DAW's capabilities were used to produce all the possible permutations of the three features being analyzed (duration, intensity, pitch), leading to the final set of 81 audio files. An example of permutation is a nonword whose pitch profile comes from the U original audio file, whose duration profile comes from the PE original file, and whose intensity profile comes from the AP original file.

The processing was composed of the following steps:

(i) The three original versions of each nonword were loaded in Cubase (Fig. A1). This software shows audio regions that are used as main carriers of the pitch information and/or as targets for duration and intensity modifications (e.g., to combine the pitch pattern of a U-stress nonword and the duration + intensity patterns of a PE nonword, the U original nonword is modified in terms of duration and intensity in order to match the features of the PE).

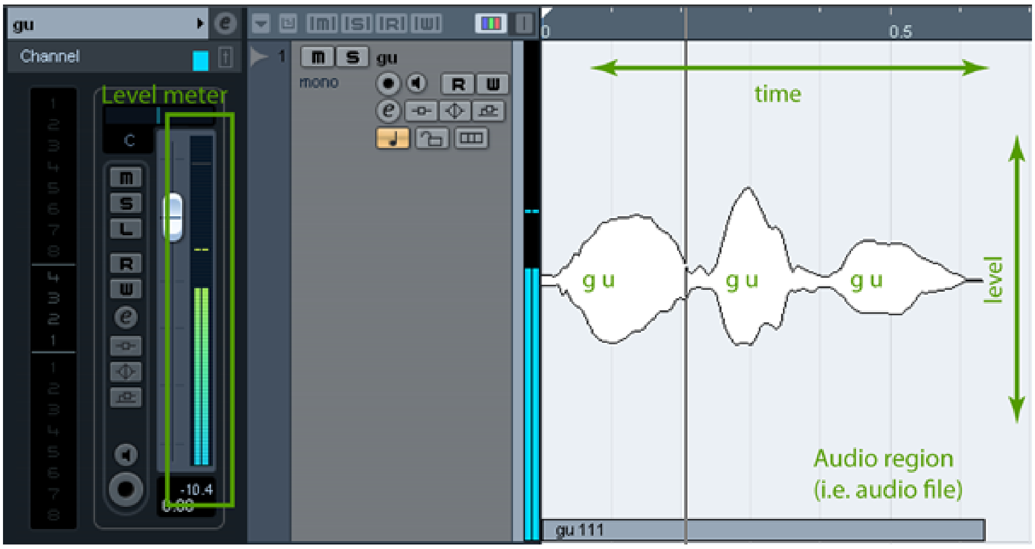

Figure A1 *An example of Cubase interface, showing the audio region, the time and level (i.e., intensity) axes, and the level meter (pitch is implicit in the audio signal).*

(ii) The *Free warp* and *ruler* tools are used to manipulate duration (Fig. A2):

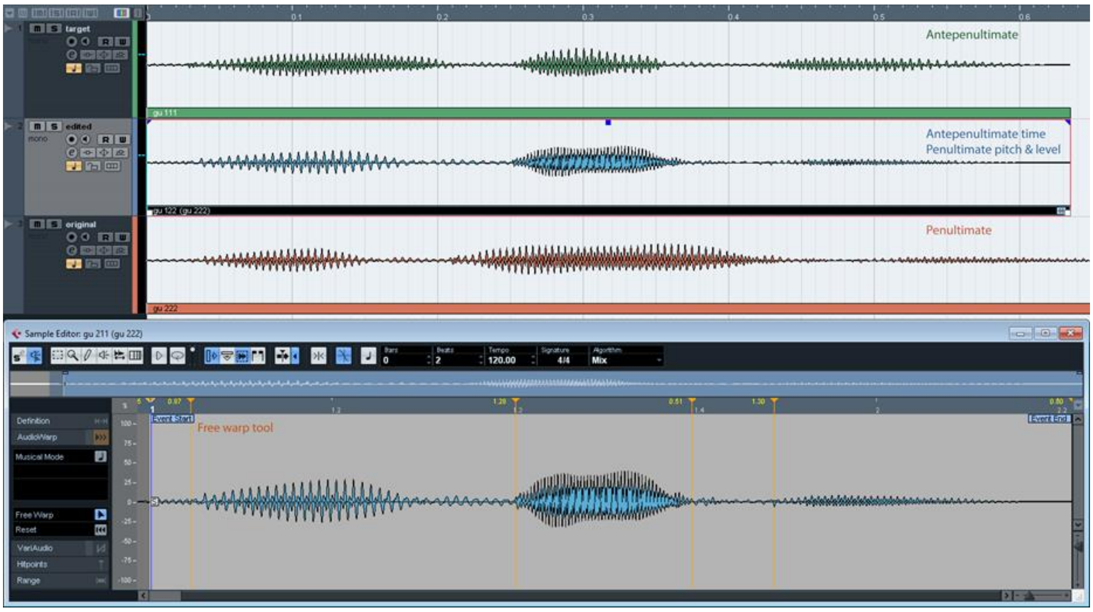

Figure A2 *Three audio regions are shown in the top window: the 'target' one reports the waveform whose timing (syllable duration pattern) needs to be copied (green); the 'edited' window shows the currently manipulated waveform (blue), and the 'original' window shown the original waveform (red). The bottom window shows the Warp editor of Cubase: orange markers can be set and shifted so that the waveform timing of the PE-stressed non-word matches the AP reference. Broad adjustments were carried out by looking at the waveform, while finer adjustments were made by looking at the time ruler on the top.*

(iii) The *Draw envelope* and *metering* tools were used to manipulate the sound intensity of each syllable (Fig. A3).

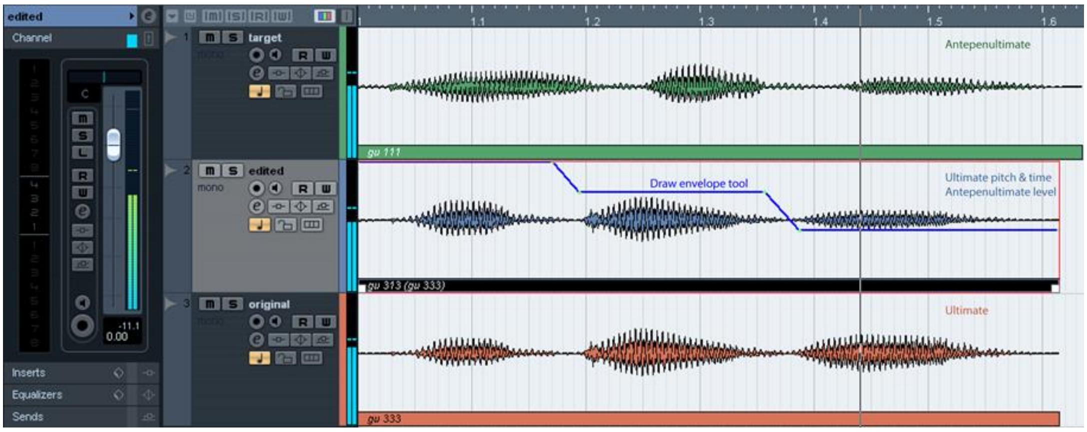

Figure A1 *Three audio regions are shown: the first ('target') reports the waveform whose intensity pattern needs to be reproduced (green), the second ('edited') reports the currently manipulated waveform (blue), and the third reports the original waveform (red). The 'edited' region also displays a blue line produced by the Draw Envelope tool, which modulates the intensity (level) of the U stimulus to match that of the AP stimulus. Broad adjustments were carried out by looking at the waveform, while finer adjustments were achieved by looking at the level meter on the left side of the window.*
